# Health Locus of Control and Medical Behavioral Interventions: Systematic Review and Recommendations

**DOI:** 10.2196/52287

**Published:** 2024-10-10

**Authors:** Sogol Mozafari, Alan Yang, Jason Talaei-Khoei

**Affiliations:** 1 Department of Information Systems College of Business University of Nevada, Reno Reno, NV United States

**Keywords:** medical behavioral interventions, health locus of control, internal control, external control, patient behavior, synthesis, review methods, literature review, narrative review, behavior change

## Abstract

**Background:**

Health locus of control (HLOC) is a theory that describes how individuals perceive different forces that influence their lives. The concept of a locus of control can affect an individual’s likelihood to commit to behaviors related to their health. This study explores the literature on the relationships between HLOC and medical behavioral interventions.

**Objective:**

This study aims to better understand how HLOC constructs can potentially affect patient responses to health behavioral interventions and to propose a series of guidelines for individuals interested in designing medical behavioral interventions related to HLOC.

**Methods:**

We used the PRISMA (Preferred Reporting Items for Systematic Reviews and Meta-Analyses) methodology and performed an analysis of 50 papers related to the topic of HLOC and medical behavioral interventions. Inclusion criteria were studies that had a behavioral intervention involving patients and contained a metric of at least 1 of the constructs related to HLOC. The initial screening and search were conducted by 2 researchers (AY and SM) separately. The results were then combined and compared.

**Results:**

Our findings explore the influence of different levels of HLOC along with the importance of both patient- and health-related context when assessing the relationships between HLOC constructs and the likelihood of health behavior change. The findings show that different constructs related to HLOC can act as reliable predictors for patient responses to medical behavioral interventions. Patients who score higher on internal HLOC measures are more likely to exhibit behaviors that are consistent with positive health outcomes. Patients who score higher on chance HLOC are more likely to exhibit behaviors that may lead to adverse health outcomes. These conclusions are supported by most of the 50 studies surveyed.

**Conclusions:**

We propose guidelines for individuals designing medical behavioral interventions so that they can make use of these relationships linked to HLOC. The three guidelines suggested are as follows: (1) in most situations, improving internal HLOC will improve health outcomes for patients; (2) patients with high external HLOC should be further studied to determine the source of the external HLOC; and (3) patients with a high chance HLOC are less likely to follow preventative behaviors or be responsive to interventions. Limitations of the study are that the primary search and analysis were conducted by 2 principal researchers (AY and SM). Interpretation and development of the guidelines are subject to individual interpretation of results and may not be applicable to all contexts.

## Introduction

### Overview

Health locus of control (HLOC) refers to an individual’s beliefs about the extent to which they have control over their health outcomes. Loci of control (LOCs) are typically split into 2 categories: internal and external forces. Individuals who have a higher internal LOC tend to believe that their actions can influence their health outcomes directly. Individuals with a higher external LOC are more likely to believe that their health outcomes are determined by external factors such as luck, fate, or the actions of other people. HLOC has been used as a theoretical model to predict the likelihood of individuals performing health-related behaviors across multiple settings. Medical behavioral interventions describe a broad range of activities; examples include the following: increasing the likelihood of individual vaccination rates through health messaging, increasing an individual’s willingness to follow a meal plan and diet by communicating the benefits of weight loss, and increasing medication adherence through targeted messaging provided by a physician to a patient.

Medical behavioral interventions are meant to affect the health behaviors of individuals with the intention of improving health outcomes. HLOC is often used as a lens to interpret how an individual interprets and internalizes health-related stimuli. Health messaging, medication efficacy, and behavioral changes are all factors that have relationships with HLOC constructs [1,2]. Understanding an individual’s HLOC can therefore be an important factor in determining the most effective approach to medical behavioral interventions.

### Research Question

The research question we are seeking to address is how do HLOC constructs affect the efficacy of health behavioral interventions? To explore this question, we conducted a systematic review of academic studies and randomized controlled trials related to health behavioral interventions and HLOC constructs. In addition to this research question, we also seek to develop a series of guidelines for individuals interested in designing medical behavioral interventions related to HLOC. The next section will provide background on the research space, specifically the HLOC and behavioral interventions in the health care context.

### Background

#### HLOC Theory

The theory of LOC was developed in the 1950s by Julian Rotter [3]. Rotter separated the LOCs into 2 constructs: internal control and external control. The distinction between the 2 constructs is the extent to which an individual believes that the outcome of an event is “contingent on their own behavior” or “a function of chance, luck, or fate” [4,5]. HLOC is an extension of the LOC theory and relates to how much control people believe they have over their health. HLOC can also be either internal or external. HLOC extends the idea of external control by creating additional constructs to better define the source of external control. Studies on external HLOC (E HLOC) typically split the construct into 3 distinct sources: powerful others HLOC (PO HLOC), chance HLOC (C HLOC), and god HLOC (G HLOC) [2,6]. Internal HLOC (I HLOC) represents the belief of individuals in their ability to impact their health status, while E HLOC represents individual belief that external sources affect personal health [7].

Table 1 [4] describes the constructs of the LOC theory we focus on in this study. Internal and external LOCs are used to distinguish the individual constructs described in the table.

**Table 1:** Locus of control.

Locus of control	Description
Internal	Individuals believe in their impact on their behavioral outcomes
External: powerful others	The belief of external sources and factors influencing one’s life and decisions
External: chance	The belief of chance and fate influence one’s life and decisions
External: god	The belief through religion of a higher power influencing one’s life and decisions

The HLOC constructs are independent of each other and can be applied to different contexts. People with higher I HLOC tend to believe in their influence on their behavioral outcomes, while individuals who have higher E HLOC believe that factors beyond their control affect those outcomes more strongly [8]. The impact of I HLOC can be effective toward physical self-care [9] and may also be an influential motivation relating to participation and the use of technologies or other tools designed for health care [5,10,11]. E HLOC and PO HLOC can be significant especially when it comes to the use of provider-recommended digital tools [1]. Individuals with I HLOC and PO HLOC beliefs may be more willing to use mobile health apps and platforms and are more likely to participate in monitoring health behaviors and developing web-based trackers [1]. HLOC has been used as a framework for analyzing individual health-related behaviors, particularly those related to platform use and trust.

HLOC remains a relevant theory for analyzing individual behavior within a health context. During the COVID-19 pandemic, many individuals experienced increases in factors related to I HLOC such as anxiety, fear, and depression. This increase led to many individuals experiencing posttraumatic stress disorder [8]. I HLOC is a commonly used framework for assessing individual factors influencing stress and developing solutions to manage anxiety [8]. E HLOC describes the extent to which an individual believes outside forces affect their life. The construct of powerful others in a health care context is usually a measurement of individual trust in a health-related platform, app, or doctor [12].

#### Medical Behavioral Interventions

Medical behavioral interventions refer to a wide range of strategies for the promotion of healthy behaviors in individual patients with the goal of improving health care outcomes [13-15]. Interventions can take on different forms including communicating health information to patients, providing incentives for desired behavior, or directly administering medication. Behavioral interventions tend to combine communication of information along with incentivization of healthy behaviors such as dieting, exercise, medication adherence, or cessation of substance abuse [16-19].

The efficacy of behavioral interventions depends on many factors. The desired health outcome and the demographics of the patient have large effects on the efficacy of different forms of interventions. For example, social support has been demonstrated to improve patient adherence to behavioral interventions related to lifestyle change such as dieting or exercise [20,21]. Interventions that are focused on acute diseases can change the efficacy of social incentives. Studies on behavioral interventions and cancer show that the severity of the disease affects adherence to guidelines delivered through an information-based health intervention [22-24]. Patients who are in the early stages of cancer tend to react strongly to new sources of information and seek out more when prompted, whereas patients in the latter stages are more receptive to the information provided by a trusted source such as a physician [25]. The consistency with which patients follow behavioral interventions is also not guaranteed. Most interventions tend to be administered over a period between 6 and 12 months. Past the intervention period, there is no certainty that patients who were receptive to the intervention messaging continued with the recommended behaviors [26-28].

In response to these issues, research has suggested that interventions tailored to specific individuals and their needs are more suitable than generalized approaches for behavioral change [29-32]. HLOC is a theory that is specific to individuals. An individual’s perception of the world and what affects their health is an indicator of how they will respond to different forms of intervention [33-35]. Exploring this avenue of research can help uncover relationships between individual perceptions and the predictive efficacy of health interventions that would otherwise be unclear.

## Methods

### Overview

To accomplish our research objectives, we approached a systematic review of the literature using the PRISMA (Preferred Reporting Items for Systematic Reviews and Meta-Analyses) methodology (Multimedia Appendix 1). PRISMA is a common methodology used to synthesize literature and filter sample pools in preparation for analysis [36-38]. Specifics regarding the search are reported below.

### PRISMA Search

We used the following terms when searching for papers related to our review: “Health Locus of Control”; “Behavior Change”; “Behavioral Change”; “Behavioral Change Interventions”; “Medication Adherence”; “HLOC”; “Patients”; “Healthcare; “Culture”; “Empirical”; and “Locus of Control.” The terms were searched individually, then altogether using the keyword of “OR” connecting all of the different terms. Finally, the terms were searched together using the keyword “AND.” These individual searches also included linked terms with “AND”: “Health Locus of Control” AND “Behavior Change”; “Health Locus of Control” AND “Behavioral Change”; “Health Locus of Control” AND “Behavioral Change Interventions.” The spelling of “Behaviour” was also used in the search for completeness. These keywords were used in a title plus abstract search through the databases of Web of Science, IEEE Xplore, the ACM Digital Library, the AIS e-Library, and PubMed. The search was conducted between November and December 2022. Additional details regarding the search and search strategy are available in Multimedia Appendix 2.

Inclusion criteria were studies that had a behavioral intervention involving patients and contained a metric of at least 1 of the constructs related to HLOC. Only studies that had actual data were considered; proposals and simulations were excluded. Additionally, only studies that were published in peer-reviewed journals were considered for inclusion. The initial screening and search were conducted by 2 researchers (AY and SM) separately. The results were then combined and compared. The initial search identified 357 papers potentially relevant to the study. Only references were used as a check for this stage, no abstracts or full texts of papers were read. After additional filtering, 245 redundant papers were removed from this pool. Reasons for paper removal during this stage included duplication due to cross-referencing and singular papers that appeared in multiple databases. Papers were then further checked for relevancy to the research scope. Filtering at this stage occurred from a reading of paper abstracts for relevance. If a study was not relevant based on a read of the abstract because it did not deal with medical behavioral intervention or HLOC or contain actual data, it was excluded. Papers that proposed studies but did not conduct behavioral interventions were also excluded at this stage. This resulted in the removal of an additional 72 papers, with 50 remaining for analysis. During this stage, citation software Zotero (Corporation for Digital Scholarship) was used to collect and compare citations and documentation.

### Data Extraction and Data Synthesis

Data extraction occurred after the identification of the items to be included in the analysis. Data extracted included the type of study, sample size, and interaction effects related to HLOC and the medical behavioral intervention. The full text of papers filtered through the first steps was read. Extraction was conducted by 2 independent researchers (AY and SM). Discrepancies were solved through a discussion of the 2 researchers and consensus was required to include information in the final document. Multimedia Appendix 3 [1,5,8,12,22,23,25,27,39-80] contains the results of this data extraction process. Data synthesis took on a similar form to extraction, 2 independent researchers (AY and SM) evaluated the information collected and discrepancies were handled through consensus decision after discussion. After collating the results from the review, we analyzed the frequencies of studies that had relationships between the primary HLOC constructs and related health care behaviors. From this analysis, we developed a series of guidelines for researchers interested in the field of patient behavioral change in health care settings.

## Results

### Overview

**Figure 1:**
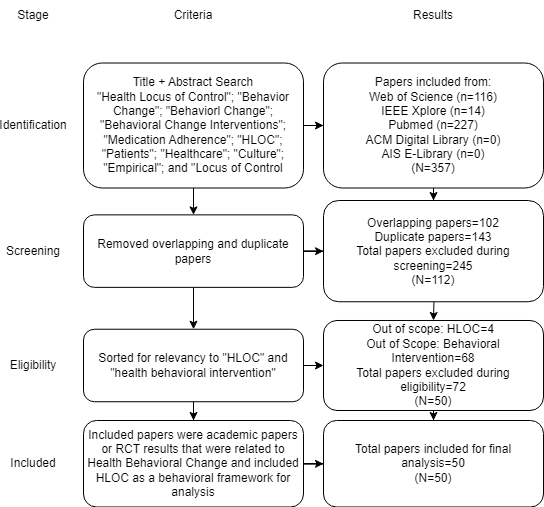
PRISMA Search Strategy and Results.

The following sections will cover the results of the data synthesis. Topics of discussion include relationships between constructs, behavioral health interventions, and the influence of contextual factors such as disease and patient characteristics observed within the studies. Figure 1 contains the PRISMA flow diagram summarizing the search process.

The main health behaviors discussed in all the papers reviewed in this study are labeled into 5 main categories. These categories are preventative health behaviors, mental health, personal health perception, vaccination hesitancy, and physician trust. The number of papers that have focused on each health behavior is shown in Table 2.

The number of HLOC constructs that have been focused on in the 50 papers reviewed for this study can be seen in Table 3. Some studies have focused on several constructs, and some have focused on only one. The following table shows the counts of HLOC constructs that are mainly highlighted in this study’s review papers.

**Table 2:** Health behaviors.

Health behavior	Papers (n=50), n (%)
Preventative health behaviors	22 (44)
Mental health	20 (40)
Personal health perception	5 (10)
Vaccination hesitancy	2 (4)
Physician trust	1 (2)

**Table 3:** Number of HLOC^a^ constructs.

HLOC constructs	Uses (n=50), n (%)
Internal HLOC	45 (90)
Powerful others HLOC	37 (74)
Chance HLOC	34 (68)
God HLOC	9 (18)

^a^HLOC: health locus of control.

### HLOC and General Health

The relationship between HLOC and general patient health depends on many different factors. However, common relationships between HLOC constructs and patient health can be observed that are generalizable across contexts. The discussion in this section will focus on the I HLOC, E HLOC, PO HLOC, and C HLOC.

Higher I HLOC generally leads to better health outcomes overall due to higher rates of patient self-efficacy. Higher patient self-efficacy leads to an increase in behaviors that are beneficial to overall patient health. These behaviors can include improved medication adherence, preventative health behaviors, and an increase in healthy activities. These behaviors and outcomes are observable across income levels and other demographic characteristics [39,40].

Higher E HLOC within individuals can have different outcomes depending on the prevailing construct: C HLOC or PO HLOC. Higher C HLOC generally leads to more negative health behaviors and health outcomes in individuals. Individuals with high C HLOC tend to believe that health is an uncontrollable outcome that is determined by chance. As a result, those individuals tend to perform fewer preventative behaviors that could improve health outcomes. PO HLOC has a less clear relationship with general health. The source of the powerful other has a large impact on the decision an individual makes. For instance, individuals with a high PO HLOC who see physicians as the primary source of powerful others are likely to adhere to medical recommendations and have better health outcomes as a result. Individuals who have similarly high PO HLOC but view friends and family as the powerful other may have mixed results, as the friends and family may not recommend behaviors that are consistent with medical recommendations.

### HLOC and Health Interventions

Research has shown that higher I HLOC can lead to more preventative behavior from patients [41]. Patients with higher PO HLOC have more potential influences [21]. In fact, those with a higher PO HLOC are more likely to engage in preventative behaviors, particularly if the influence comes from physicians or other health care professionals [42]. However, if the PO HLOC comes from religion or a belief in a higher power, health care behaviors are likely more context dependent and less predictable [43]. For example, a high PO HLOC sourced to a patient’s family may cause contradictory health outcomes such as lower vaccination rates, higher social contact, and higher physical activity. On the other hand, those with a higher C HLOC, or the belief that health outcomes are beyond their control, are less likely to engage in preventative health behaviors, often believing that their efforts won’t matter.

Studies have found that individuals with higher I HLOC are more likely to engage in healthy dietary behaviors. This includes less consumption of unhealthy foods and more intake of healthy foods. On the other hand, those with higher C HLOC are less likely to adopt beneficial dietary patterns [44]. Interestingly, individuals with higher PO HLOC tend to have more beneficial dietary patterns. For example, parents can be seen as power others, and if an individual has a higher PO HLOC, they may be more likely to adopt a healthy diet based on their parents’ influence [44]. Overall, an individual’s HLOC plays a significant role in their dietary behaviors and can be a useful factor to consider when developing effective interventions for improving nutrition.

When it comes to substance abuse, research suggests that HLOC can play a significant role in this context. Individuals with higher I HLOC tend to make self-centered decisions that can lead to reduced substance abuse. This includes decisions such as smoking reduction, birth control use, and weight loss [9]. Additionally, those with lower I HLOC are less likely to make these self-centered decisions and tend to act more carelessly toward their health [45]. Examples would be more alcohol consumption and daily smoking. Similarly, individuals with a higher C HLOC have been found to be more likely to smoke. Conversely, those with a higher PO HLOC are less likely to smoke due to the influence of their family or physicians [45]. Understanding an individual’s HLOC can be an important factor in developing effective interventions to reduce substance abuse and improve overall health outcomes.

### Additional Factors

Besides the individual contexts of the relationships between constructs, individual patient characteristics can also affect how HLOC constructs impact health behaviors. Patients who have chronic conditions tend to have higher C HLOC, particularly over longer periods. The severity of illnesses also affects the usual relationships between HLOC constructs and patient health. In the context of chronic conditions, such as asthma, patients with higher self-efficacy and higher I HLOC are in more control of their condition, whereas patients with higher E HLOC beliefs can potentially lead to poorer control [46].

### Patient Characteristics

Several factors have been found to influence HLOC, including social contact, health information–seeking behavior, and trust in health care providers. For example, social contact has been found to lead to better health care behaviors across all income levels [39]; higher I HLOC has been found to lead to more social behaviors, which in turn leads to positive health influence [47]; and health information–seeking behavior has been linked to higher I HLOC, indicating individuals with higher I HLOC are more likely to seek out information to be well aware of their health decisions outcome [39]. Higher E HLOC, and mostly higher PO HLOC, can increase patients’ level of trust, and patients who are more trusting have been found to be more likely to follow their physician recommendations [12]. C HLOC has also been found to have a moderating effect on patient self-efficacy and self-management behavior [48]. High self-efficacy leads to more self-management, and depending on the level of patients’ C HLOC, this relationship can be either strengthened or weakened.

E HLOC has been found to affect death-related anxiety. Higher PO HLOC can lead to less stress regarding the uncontrollable nature of death based on the trust in others’ (physicians’) influence on one’s health outcomes [49]. However, higher C HLOC has been found to increase the level of anxiety and depression at the end of life, as it indicates patients’ lack of control over their health and the inevitability of death [22]. These patterns are also applicable to healthy patients as they age (Aviad and Cohen-Louck [72]). E HLOC can also impact patients both positively and negatively. An elevated E HLOC can result in an increased level of depression and anxiety for a patient. For example, patients with high E HLOC have been found to have poor coping skills, which could result in higher levels of depression. Within the same context, patients with high E HLOC linked to caregivers are more likely to rely on their physician, worry less, and adapt to their conditions [22,81].

### Caregiver Characteristics

The impact of an individual’s health decisions on other individuals and their relationship with them can also affect their perceived HLOC. For example, if a patient is concerned about their child’s health outcome, the parent’s I HLOC and E HLOC may be influenced by their perception of their ability and connected anxiety related to their child’s health outcomes. For example, parental caregiving quality increases when a child faces challenges such as a chronic condition [27]. Some studies have found mothers to be the main caregivers in special chronic conditions such as type 1 diabetes [82,83]. Parents of children dealing with diabetes were found to have higher I HLOC, whereas patients who had failed to reach that control level tended to have lower levels of I HLOC and higher levels of G HLOC and C HLOC [82]. Mothers and other caregivers have also been found to influence patients’ behavior and coping styles. The level of I HLOC or E HLOC of the caregiver can significantly impact the patients’ active or passive coping style and how they behave toward their condition and perceived anxiety level [84].

### Disease Characteristics

Terminal illnesses can lead to unique cases where higher I HLOC causes greater stress and anxiety for patients as they experience a greater loss of control [22,50]. The relationship between these 2 constructs is stronger as the illness becomes more serious. A reversal of this relationship is possible. Patients who are provided with an option for treatment or hopeful messaging can decrease stress and anxiety among patients with higher I HLOC. The likely reason for this phenomenon is that patients with higher I HLOC perceive a return of control, which leads to lower stress and more proactive behaviors when positive messaging is provided.

Patients with cancer across all stages tend to have higher PO HLOC and decreased I HLOC. Higher PO HLOC tends to manifest in a lowering of anxiety if the source of the powerful other is rooted in religion or part of the G HLOC [25]. The reverse of this relationship has also been observed. Patients with cancer with higher I HLOC tend to have lower E HLOC. The higher I HLOC is usually indicative of a higher quality of life for the duration of the disease [51]. This might be due to the impact of I HLOC in the hope of recovery, which leads the patient to a better mental state and lowers the anxiety and depression rates.

Uncertainty is a major consideration across multiple studies focused on patient behaviors. Uncertainty is defined as any situation where a decision maker is unable to accurately predict outcomes because they lack information or sufficient environmental cues [85]. In the concept of HLOC, individuals with higher I HLOC are more likely to react to uncertainty as a controllable decision and opportunity, whereas individuals with higher E HLOC may consider their uncertainty as untrustworthy evidence of a situation [86]. Patients with a higher level of I HLOC tend to be more involved in their health-related situations and have less uncertainty about them [51,87,88]. Higher I HLOC and more level of uncertainty in patients can result in less stress and anxiety rates.

Research shows that uncertainty mediates between HLOC with quality of life, anxiety, and depression [51]. It has been illustrated that the level of a patient’s education and the complexity of a treatment can directly affect patients’ uncertainty which can be reduced by improving the level of education and patients’ knowledge about the related phenomenon [89-91]. Physicians and health professionals can be external resources for providing such information to patients with higher PO HLOC and help them overcome their uncertainty and improve their mental health [91,92]. Patients with higher I HLOC have been found to have better coping with their condition and adapt well to the change, along with information-seeking behavior, which would result in improved level of knowledge about their condition and reduced level of stress [51,87]. Additionally, patients who have dealt with chronic conditions for longer periods feel less uncertainty and less stress about their condition because of their perceived knowledge about their situation over time which would result in their improved quality of life and mental health. With chronic conditions, such as asthma, patients with higher self-efficacy and higher I HLOC are in more control of their condition, whereas patients with higher E HLOC beliefs can potentially lead to poorer control [46]. In the case of patients with dementia, higher E HLOC has been observed with lower patient depression, which could be because E HLOC may be linked to acceptance of chronic conditions (Halse et al [50]).

## Discussion

### Principal Findings

Through analysis of the data, we observed intersections between HLOC characteristics of patients and health outcomes. The most commonly recurrent themes are related to I HLOC, E HLOC, and C HLOC. The effects of these constructs varied across studies. Based on common patterns observed across the data, we offer the following suggestions for individuals designing medical behavioral interventions. First, in most situations, improving I HLOC will improve health outcomes for patients. I HLOC has been observed to improve patient health outcomes across many different contexts. Patients with higher I HLOC feel a greater sense of ownership over their health, which leads them to take more proactive measures and makes them more receptive to health behavioral interventions. The one exception to this guideline is situations where a patient’s health condition is terminal and an individual has limited control over the outcome. In such situations, high I HLOC can be problematic and lead to higher stress in contexts where patients feel a loss of control [88]. In these situations, cultivating E HLOC or G HLOC or minimizing I HLOC through a discussion around acceptance of outcomes and a release of personal control may be beneficial [51]. Second, patients with high E HLOC should be further studied to determine the source of the E HLOC. A common misconception of patients with high E HLOC is that they are not as receptive to health messaging because they are not intrinsically motivated like individuals with high I HLOC. Individuals with high E HLOC can be just as receptive and proactive as individuals with high I HLOC, but the source of the external influence is an important factor to consider. Patients who perceive their physicians or similar caregivers as the source of the E HLOC will be more receptive to following suggestions provided by the perceived authority [3]. However, patients who identify their main E HLOC source as friends, family, or media may hold beliefs that are contradictory to positive health messaging [52]. Determining the source of E HLOC is crucial to a better understanding of how those patients are likely to respond to health interventions. Third, patients with high C HLOC are less likely to follow prepreventative behaviors or be responsive to interventions. Across all studies, contexts, and situations surveyed for this review, patients with higher C HLOC were less prone to be proactive about their health. This likely stems from the idea common to individuals with demonstrable high C HLOC that actions are ultimately meaningless because everything happens through chance including health outcomes. To address these issues, health interventionalists should identify patients who exhibit high C HLOC and seek to educate them on the benefits of behavioral change. Demonstrations on the effectiveness of interventions can also help to decrease C HLOC and improve healthy patient behaviors.

### Comparison to Prior Work

We explored the relationship between HLOC and health behavioral interventions in this paper. Previous studies have explored similar relationships related to individual-level constructs and responses to health behavioral interventions and health messaging. In this section, we explore some of the prior work in the literature and how it relates to individual-level beliefs regarding personal health.

HLOC and more generally LOC as a whole pertain to a high degree of conceptual similarities with the attributional notion of locus of cause, which refers to individuals’ perception of their causation [93]. The concepts, however, differ in the way a specific behavior is observed: either from an outside observer’s perspective or one observing his or her behavior [94]. The locus of cause is considered internal when someone can perform a task and tries to do so. In this case, failing the task is attributed to a lack of trying and effort of the person. In contrast, when someone does not have the required abilities for a specific task, the locus of cause appears to be external, and the related failure is assumed to be due to external circumstances [93,94]. Similarly, the theory of self-attribution bias highlights a situation in which individuals excessively attribute credit to their abilities for past successes, while assigning blame to others for failures [95-97]. Based on this concept, individuals attribute themselves to positive outcomes, linking them to their actions and efforts, while ascribing negative results to external factors such as bad luck [98]. In the context of LOC, individuals either perceive themselves to have the ability to control an outcome and try to do so or perceive themselves as not having the ability to control an outcome and therefore do not put any effort into achieving that. It can be concluded that the LOC can be simplified as a way of attributing the cause and outcomes of one’s actions to oneself [94].

Studies have posited that older people and those with lower levels of education have higher levels of E HLOC beliefs over internal ones [40]. In addition to education, various studies have reported higher E HLOC in individuals with low sociodemographic status or negative health-related behavior such as smoking and drinking [1,99-104]. This association has been specifically investigated by Wallston and Wallston [105], which highlighted higher PO HLOC and higher C HLOC in individuals with less than 12 years of education. Conversely, higher I HLOC can be seen in individuals with high socioeconomic status or positive health behavior such as regular exercising or staying on a healthy diet [53]. On the same stream of thoughts, the HLOC theory has been considered as a possible mediator between socioeconomic status and health outcomes [53,106].

### Limitations and Strengths of the Systematic Review

The systematic review has inherent limitations. Observation of a field of research is dependent upon the scope and perception of the researchers cataloging the information. Analysis and interpretation of results are affected by subjective factors. In this paper, we have attempted to minimize individual biases and subjectivity by establishing inclusion and exclusion criteria and having sourcing and analysis of data conducted by 2 researchers (AY and SM) independently. The strengths of the systematic review are the ability to provide a comprehensive view of the state of research and the ability to generate guidelines that are related to current trends and recent phenomena in the field.

### Future Work

This study focused on the literature surrounding HLOC constructs and health behavioral interventions. New health behavioral interventions related to the COVID-19 pandemic and the subsequent policies offer new avenues for the exploration of HLOC and individual health behaviors. Future studies could explore how social distancing, vaccinations, or quarantining is related to the idea of HLOC and the propensity of individuals to adhere to guidelines. In general, it is probable for individuals with higher PO HLOC to be influenced by other individuals’ suggestions. However, individuals with higher I HLOC are also likely to get this influence from others depending on the situation. A relative example of this context is the nonforcing manipulation through nudges. Nudging entails organizing and modifying a decision-making scenario without restricting choice options or imposing force, in a way to alter someone’s behavior in a predictive way [107]. For instance, a smoker with high I HLOC may decide to quit smoking due to a close friend telling him or her about the infertility risks caused by smoking. In this example, the individual’s decision about willingly stopping an internal choice with the intention of maintaining his or her health is taking place because of someone else’s nudge. In other words, this person believes in having self-control over this decision and is aware of having the choice of not quitting as well but is still influenced to do so. Many examples can be applicable in this context, depending on the specific scenario and individuals’ circumstances. We recommend future research to expand on the impact of “compelling versus noncompelling manipulation,” as well as “effective nudges from friends or family versus strangers” on HLOC.

An exploration of the individual sources of E HLOC can also be beneficial. E HLOC is often interpreted in a similar manner to I HLOC. However, the sources of E HLOC are myriad and can vary from individual to individual. More studies that explore how changes to different HLOC constructs affect patient behaviors over time would support the relationships observed in many of the papers. One additional avenue of research is the implementation of health interventions with artificial intelligence. Research on this topic has already explored the possibility of integrating language models or similar generative agents with adaptive responses to improve patient health outcomes [108,109]. The guidelines suggested in this paper could also be tested in a longitudinal study where changes in HLOC construct strength could be measured against patient behaviors in response to behavioral interventions.

This study’s methodology is a comprehensive literature review. Studies were filtered and analyzed based on relevance but there was no metric applied to rate the quality of the studies or to determine the suitability of the findings beyond the existence of data collection and analysis. Future studies could apply more stringent metrics toward inclusion of studies, such as by omitting studies that do not exceed a certain sample size or only including studies that meet a particular duration.

### Conclusions

The study of HLOC and medical behavioral interventions is the study of how each individual responds to messaging and motivations regarding their health-related behaviors. An increased understanding of the constructs and relationships across these 2 ideas can lead to better-designed studies and interventions across different populations. Further exploration of this topic can focus on the importance of individual characteristics and the influence of context on each HLOC construct. The patterns and relationships frequently observed can help both academics and practitioners better design studies to explore questions related to improving health outcomes. Better-designed interventions can lead to individuals taking a more active role in managing their health, ultimately leading to improved health outcomes for everyone.

## Appendix

Multimedia Appendix 1 Preferred Reporting Items for Systematic Reviews and Meta-Analyses extension for Scoping Reviews (PRISMA-ScR) checklist for systematic literature review.

Multimedia Appendix 2 Search strategy for systematic literature review.

Multimedia Appendix 3 Notes for systematic literature review.

## Abbreviations

C HLOC: chance health locus of control
E HLOC: external health locus of control
G HLOC: god health locus of control
HLOC: health locus of control
I HLOC: internal health locus of control
LOC: locus of control
PO HLOC: power others health locus of control
PRISMA: Preferred Reporting Items for Systematic Reviews and Meta-Analyses

## References

[1] Bennett BL, Goldstein CM, Gathright EC, Hughes JW, Latner JD (2017). Internal health locus of control predicts willingness to track health behaviors online and with smartphone applications. Psychol Health Med.

[2] Kassianos AP, Symeou M, Ioannou M (2016). The health locus of control concept: factorial structure, psychometric properties and form equivalence of the multidimensional health locus of control scales. Health Psychol Open.

[3] Rotter J (2011). Rotter internal-external locus of control scale. 28 Meas Locus Control.

[4] Rotter JB (1966). Generalized expectancies for internal versus external control of reinforcement. Psychol Monogr Gen Appl.

[5] Ahadzadeh AS, Wu SL, Ong FS, Deng R (2021). The mediating influence of the unified theory of acceptance and use of technology on the relationship between internal health locus of control and mobile health adoption: cross-sectional study. J Med Internet Res.

[6] Wallston KA, Wallston BS, DeVellis R (1978). Development of the multidimensional health locus of control (MHLC) scales. Health Educ Monogr.

[7] Wallston KA (2005). The validity of the multidimensional health locus of control scales. J Health Psychol.

[8] Chandra Y, Yagnik J (2022). Experience of perceived stress and impact of health locus of control during COVID-19 pandemic: investigating entrepreneurs and corporate employees. South Asian J Hum Resour Manag.

[9] Wallston BD, Wallston KA (1978). Locus of control and health: a review of the literature. Health Educ Monogr.

[10] Ryan RM, Deci EL (2000). Self-determination theory and the facilitation of intrinsic motivation, social development, and well-being. Am Psychol.

[11] Yang A, Varshney U (2022). Mobile health evaluation: taxonomy development and cluster analysis. Healthc Anal.

[12] Hillen MA, de Haes HCJM, Stalpers LJA, Klinkenbijl JHG, Eddes EH, Verdam MGE, Smets EMA (2014). How attachment style and locus of control influence patients' trust in their oncologist. J Psychosom Res.

[13] Lippke S, Ziegelmann JP (2008). Theory-based health behavior change: developing, testing, and applying theories for evidence-based interventions. Appl Psychol.

[14] Ryan P (2009). Integrated theory of health behavior change: background and intervention development. Clin Nurse Spec.

[15] Whittaker R (2012). Issues in mHealth: findings from key informant interviews. J Med Internet Res.

[16] Lu Y, Stathopoulou T, Vasiloglou MF, Pinault LF, Kiley C, Spanakis EK, Mougiakakou S (2020). goFOODTM: an artificial intelligence system for dietary assessment. Sensors (Basel).

[17] MacPherson MM, Merry KJ, Locke SR, Jung ME (2019). Effects of mobile health prompts on self-monitoring and exercise behaviors following a diabetes prevention program: secondary analysis from a randomized controlled trial. JMIR Mhealth Uhealth.

[18] Nabovati E, Vakili-Arki H, Taherzadeh Z, Saberi MR, Medlock S, Abu-Hanna A, Eslami S (2017). Information technology-based interventions to improve drug-drug interaction outcomes: a systematic review on features and effects. J Med Syst.

[19] Borghouts J, Eikey E, Mark G, de Leon C, Schueller SM, Schneider M, Stadnick N, Zheng K, Mukamel D, Sorkin DH (2021). Barriers to and facilitators of user engagement with digital mental health interventions: systematic review. J Med Internet Res.

[20] Koufopoulos JT, Conner MT, Gardner PH, Kellar I (2016). A web-based and mobile health social support intervention to promote adherence to inhaled asthma medications: randomized controlled trial. J Med Internet Res.

[21] Marr J, Wilcox S (2015). Self-efficacy and social support mediate the relationship between internal health locus of control and health behaviors in college students. Am J Health Educ.

[22] Brown AJ, Thaker PH, Sun CC, Urbauer DL, Bruera E, Bodurka DC, Ramondetta LM (2017). Nothing left to chance? The impact of locus of control on physical and mental quality of life in terminal cancer patients. Support Care Cancer.

[23] Lima MP, Moret-Tatay C, Irigaray TQ (2022). Locus of control, personality and depression symptoms in cancer: testing a moderated mediation model. Clin Psychol Psychother.

[24] Hewitt S, Sephton R, Yeowell G (2020). The effectiveness of digital health interventions in the management of musculoskeletal conditions: systematic literature review. J Med Internet Res.

[25] Iskandarsyah A, de Klerk C, Suardi DR, Sadarjoen SS, Passchier J (2014). Health locus of control in Indonesian women with breast cancer: a comparison with healthy women. Asian Pac J Cancer Prev.

[26] Kang Y (2009). Role of health locus of control between uncertainty and uncertainty appraisal among patients with atrial fibrillation. West J Nurs Res.

[27] Tsiouli E, Pavlopoulos V, Alexopoulos EC, Chrousos G, Darviri C (2014). Short-term impact of a stress management and health promotion program on perceived stress, parental stress, health locus of control, and cortisol levels in parents of children and adolescents with diabetes type 1: a pilot randomized controlled trial. Explore (NY).

[28] Orlowski SK, Lawn S, Venning A, Winsall M, Jones GM, Wyld K, Damarell RA, Antezana G, Schrader G, Smith D, Collin P, Bidargaddi N (2015). Participatory research as one piece of the puzzle: a systematic review of consumer involvement in design of technology-based youth mental health and well-being interventions. JMIR Hum Factors.

[29] Hesse BW, Nelson DE, Kreps GL, Croyle RT, Arora NK, Rimer BK, Viswanath K (2005). Trust and sources of health information: the impact of the Internet and its implications for health care providers: findings from the first health information national trends survey. Arch Intern Med.

[30] Hill TD, Upenieks L, Ellison CG, Angel JL, Ortega ML, Robledo LMG (2021). Religious involvement, health locus of control, and sleep disturbance: a study of older Mexican Americans. Understanding the Context of Cognitive Aging: Mexico and the United States.

[31] Xie H, Prybutok G, Peng X, Prybutok V (2020). Determinants of trust in health information technology: an empirical investigation in the context of an online clinic appointment system. Int J Human-Computer Interact.

[32] Perez-Ramos JG, McIntosh S, Barrett ES, Velez Vega CM, Dye TD (2021). Attitudes toward the environment and use of information and communication technologies to address environmental health risks in marginalized communities: prospective cohort study. J Med Internet Res.

[33] Marcolino MS, Oliveira JAQ, D'Agostino M, Ribeiro AL, Alkmim MBM, Novillo-Ortiz D (2018). The impact of mHealth interventions: systematic review of systematic reviews. JMIR Mhealth Uhealth.

[34] Campbell RJ, Nolfi DA (2005). Teaching elderly adults to use the internet to access health care information: before-after study. J Med Internet Res.

[35] Howell P, Abdelhamid M (2023). Protection motivation perspective regarding the use of COVID-19 mobile tracing apps among public users: empirical study. JMIR Form Res.

[36] Pinho S, Cruz M, Ferreira F, Ramalho A, Sampaio R (2021). Improving medication adherence in hypertensive patients: a scoping review. Prev Med.

[37] Singh N, Varshney U (2019). IT-based reminders for medication adherence: systematic review, taxonomy, framework and research directions. Eur J Inf Syst.

[38] Mavragani A (2020). Infodemiology and infoveillance: scoping review. J Med Internet Res.

[39] Musich S, Wang SS, Slindee L, Kraemer S, Yeh CS (2020). The impact of internal locus of control on healthcare utilization, expenditures, and health status across older adult income levels. Geriatr Nurs.

[40] Poortinga W, Dunstan FD, Fone DL (2008). Health locus of control beliefs and socio-economic differences in self-rated health. Prev Med.

[41] Bianchi D, Lonigro A, Norcia AD, Tata DD, Pompili S, Zammuto M, Cannoni E, Longobardi E, Laghi F (2022). A model to understand COVID-19 preventive behaviors in young adults: health locus of control and pandemic-related fear. J Health Psychol.

[42] Marton G, Pizzoli SFM, Vergani L, Mazzocco K, Monzani D, Bailo L, Pancani L, Pravettoni G (2021). Patients' health locus of control and preferences about the role that they want to play in the medical decision-making process. Psychol Health Med.

[43] Olagoke AA, Olagoke OO, Hughes AM (2021). Intention to vaccinate against the novel 2019 coronavirus disease: the role of health locus of control and religiosity. J Relig Health.

[44] Duplaga M, Grysztar M (2021). Nutritional behaviors, health literacy, and health locus of control of secondary schoolers in Southern Poland: a cross-sectional study. Nutrients.

[45] Mercer DA, Ditto B, Lavoie KL, Campbell T, Arsenault A, Bacon SL (2018). Health locus of control is associated with physical activity and other health behaviors in cardiac patients. J Cardiopulm Rehabil Prev.

[46] Schreitmüller J, Loerbroks A (2020). The role of self-efficacy and locus of control in asthma-related needs and outcomes: a cross-sectional study. J Asthma.

[47] Kesavayuth D, Poyago-Theotoky J, Tran DB, Zikos V (2020). Locus of control, health and healthcare utilization. Econ Model.

[48] Wang R, Zhou C, Wu Y, Sun M, Yang L, Ye X, Zhang M (2022). Patient empowerment and self-management behaviour of chronic disease patients: a moderated mediation model of self-efficacy and health locus of control. J Adv Nurs.

[49] Akanni O, Koleoso O (2022). Self-esteem, locus of control and religiosity in predicting death anxiety among students in a Nigerian tertiary institution. J Clin Sci Res.

[50] Halse I, Bjørkløf GH, Engedal K, Selbæk Geir, Barca ML (2021). One-year change in locus of control among people with dementia. Dement Geriatr Cogn Dis Extra.

[51] Sharif SP (2017). Locus of control, quality of life, anxiety, and depression among Malaysian breast cancer patients: the mediating role of uncertainty. Eur J Oncol Nurs.

[52] Boyd JM, Wilcox S (2020). Examining the relationship between health locus of control and god locus of health control: is god an internal or external source?. J Health Psychol.

[53] Kuwahara A, Nishino Y, Ohkubo T, Tsuji I, Hisamichi S, Hosokawa T (2004). Reliability and validity of the multidimensional health locus of control scale in Japan: relationship with demographic factors and health-related behavior. Tohoku J Exp Med.

[54] Ganjoo M, Farhadi A, Baghbani R, Daneshi S, Nemati R (2021). Association between health locus of control and perceived stress in college student during the COVID-19 outbreak: a cross-sectional study in Iran. BMC Psychiatry.

[55] Lee DJ, So WY, Lee SM (2021). The relationship between Korean adolescents' sports participation, internal health locus of control, and wellness during COVID-19. Int J Environ Res Public Health.

[56] Shin S, Lee E (2021). Relationships among the internal health locus of control, mental health problems, and subjective well-being of adults in South Korea. Healthcare (Basel).

[57] Imeri H, Holmes E, Desselle S, Rosenthal M, Barnard M (2023). A survey study of adults with chronic conditions: examining the correlation between patient activation and health locus of control. Chronic Illness.

[58] Mehta R, Narayanan M (2021). The relationship of emotional reactivity with health locus of control. J Psychosocial Res.

[59] Aviad-Wilchek Y (2019). Locus of control and the meaning of life as a salutogenic model that reduces suicidal tendencies in patients with mental illness. Curr Psychol.

[60] Wrightson KJ, Wardle J (1997). Cultural variation in health locus of control. Ethn Health.

[61] Reknes G, Visockaite A, Liefooghe A, Lovakov A, Einarsen SV (2019). Locus of control moderates the relationship between exposure to bullying behaviors and psychological strain. Front Psychol.

[62] Mahmoud AB, Reisel WD, Fuxman L, Hack‐Polay D (2021). Locus of control as a moderator of the effects of COVID‐19 perceptions on job insecurity, psychosocial, organisational, and job outcomes for MENA region hospitality employees. Eur Manag Rev.

[63] Helmer SM, Krämer A, Mikolajczyk RT (2012). Health-related locus of control and health behaviour among university students in North Rhine Westphalia, Germany. BMC Res Notes.

[64] Amit Aharon A, Nehama H, Rishpon S, Baron-Epel O (2018). A path analysis model suggesting the association between health locus of control and compliance with childhood vaccinations. Hum Vaccin Immunother.

[65] Morishita M, Hattori S, Miyai N (2017). Ability for self-care among elderly patients with diabetes mellitus and its association with health locus of control and social support. Nihon Eiseigaku Zasshi.

[66] Kaynak H, Turan A, Demir Y (2024). Locus of control as a mediator of the relationships between motivational systems and trait anxiety. Psychol Rep.

[67] Krampe H, Danbolt LJ, Haver A, Stålsett G, Schnell T (2021). Locus of control moderates the association of COVID-19 stress and general mental distress: results of a Norwegian and a German-speaking cross-sectional survey. BMC Psychiatry.

[68] Micheletto V, Zito M, Bustreo M, Gabrielli G, Circi R, Russo V (2022). The impact of optimism and internal locus of control on workers’ well-being, a multi-group model analysis before and during the COVID-19 pandemic. Social Sci.

[69] Mori M, Seko T, Ogawa S (2022). Association of social capital and locus of control with perceived health during the COVID-19 pandemic in Japan. Int J Environ Res Public Health.

[70] Weinhardt CB, Ruckert JH (2023). Internal locus of control predicts proenvironmental and COVID-19 health-related behaviors: a pilot study. Ecopsychology.

[71] Würtzen H, Clausen LH, Andersen PB, Santini ZI, Erkmen J, Pedersen HF (2022). Mental well-being, health, and locus of control in Danish adults before and during COVID-19. Acta Neuropsychiatr.

[72] Aviad Y, Cohen-Louck K (2021). Locus of control and purpose in life as protective factors against the risk for suicide in older adults. Smith Coll Stud Social Work.

[73] Gore JS, Griffin DP, McNierney D (2016). Does internal or external locus of control have a stronger link to mental and physical health?. Psychol Stud.

[74] Grotz M, Hapke U, Lampert T, Baumeister H (2011). Health locus of control and health behaviour: results from a nationally representative survey. Psychol Health Med.

[75] Moshki M, Amiri M, Khosravan S (2012). Mental health promotion of Iranian university students: the effect of self-esteem and health locus of control. J Psychiatr Ment Health Nurs.

[76] Holroyd EA, Molassiotis A, Taylor-Pilliae RE (2001). Filipino domestic workers in Hong Kong: health related behaviors, health locus of control and social support. Women Health.

[77] Cohen M, Azaiza F (2007). Health-promoting behaviors and health locus of control from a multicultural perspective. Ethn Dis.

[78] Pieterse AL, Carter RT (2010). An exploratory investigation of the relationship between racism, racial identity, perceptions of health, and health locus of control among Black American women. J Health Care Poor Underserved.

[79] Egan JT, Leonardson G, Best LG, Welty T, Calhoun D, Beals J (2009). Multidimensional health locus of control in American Indians: the strong heart study. Ethn Dis.

[80] van Dijk TK, Dijkshoorn H, van Dijk A, Cremer S, Agyemang C (2013). Multidimensional health locus of control and depressive symptoms in the multi-ethnic population of the Netherlands. Soc Psychiatry Psychiatr Epidemiol.

[81] Broers S, Kaptein AA, Le Cessie S, Fibbe W, Hengeveld MW (2000). Psychological functioning and quality of life following bone marrow transplantation: a 3-year follow-up study. J Psychosom Res.

[82] Franceschi R, Canale M, Piras EM, Galvagni L, Vivori C, Cauvin V, Soffiati M, Maines E (2022). Influence of parental health locus of control on behavior, self-management and metabolic control, in pediatric patients with type 1 diabetes. J Pers Med.

[83] Malerbi FEK, Negrato CA, Gomes MB, Brazilian Type 1 Diabetes Study Group (BrazDiab1SG) (2012). Assessment of psychosocial variables by parents of youth with type 1 diabetes mellitus. Diabetol Metab Syndr.

[84] Polizzi C, Perricone G, Fontana V, D'Angelo P, Jankovic M, Nichelli F, Taormina C, Burgio S (2020). The relation between maternal locus of control and coping styles of pediatric leukemia patients during treatment. Pediatr Rep.

[85] Mishel MH, Clayton MF (2008). Theories of uncertainty in illness. Middle Range Theory for Nursing. 3rd Edition.

[86] Mishel MH (1988). Uncertainty in illness. Image J Nurs Sch.

[87] Lin CC, Tsay HF (2005). Relationships among perceived diagnostic disclosure, health locus of control, and levels of hope in Taiwanese cancer patients. Psychooncology.

[88] Watson M, Pruyn J, Greer S, van den Borne B (1990). Locus of control and adjustment to cancer. Psychol Rep.

[89] Ko NY, Hsu ST (2005). Informational needs, health locus of control and uncertainty among women hospitalized with gynecological diseases. Chang Gung Med J.

[90] Mishel MH (1990). Reconceptualization of the uncertainty in illness theory. Image J Nurs Sch.

[91] Mishel MH (1997). Uncertainty in acute illness. Annu Rev Nurs Res.

[92] van der Molen B (1999). Relating information needs to the cancer experience: 1. Information as a key coping strategy. Eur J Cancer Care (Engl).

[93] Heider F (1958). The psychology of interpersonal relations. Psychology Press.

[94] Stewart JE (2008). Locus of control and self-attribution as mediators of hazardous attitudes among aviators: a review and suggested applications. Int J Appl Aviat Stud.

[95] Campbell WK, Sedikides C (1999). Self-threat magnifies the self-serving bias: a meta-analytic integration. Rev Gen Psychol.

[96] Miller DT, Ross M (1975). Self-serving biases in the attribution of causality: fact or fiction?. Psychol Bull.

[97] Zuckerman M (1979). Attribution of success and failure revisited, or: the motivational bias is alive and well in attribution theory. J Pers.

[98] Stewart JE (2006). Locus of control, attribution theory, and the "Five Deadly Sins" of aviation. U.S. Army Research Institute for the Behavioral and Social Sciences.

[99] Calnan M (1989). Control over health and patterns of health-related behaviour. Soc Sci Med.

[100] Fleming MF, Barry KL (1991). Health locus of control in a primary care sample of alcoholics and nonalcoholics. Behav Med.

[101] Paxton SJ, Sculthorpe A (2007). Weight and health locus of control beliefs in an Australian community sample. Psychol Health.

[102] Schank MJ, Lawrence DM (1993). Young adult women: lifestyle and health locus of control. J Adv Nurs.

[103] Slenker SE, Price JH, O'Connell JK (1985). Health locus of control of joggers and nonexercisers. Percept Mot Skills.

[104] Stuart K, Borland R, McMurray N (1994). Self-efficacy, health locus of control, and smoking cessation. Addict Behav.

[105] Wallston KA, Wallston BS (1981). Health locus of control scales. Res Locus Control Constr.

[106] Acosta MSV, Chapman P, Bigelow PL, Kennedy C, Buchan RM (2005). Measuring success in a pesticide risk reduction program among migrant farmworkers in Colorado. Am J Ind Med.

[107] Hausman DM, Welch B (2010). Debate: to nudge or not to nudge. J Polit Philos.

[108] Kim J, Lee D, Park E (2021). Machine learning for mental health in social media: bibliometric study. J Med Internet Res.

[109] Asan O, Choudhury A (2021). Research trends in artificial intelligence applications in human factors health care: mapping review. JMIR Hum Factors.

