# Supplementary material for: Health Locus of Control and Medical Behavioral Interventions: Systematic Review and Recommendations
Source: Interact J Med Res. 2024 Oct 10;13:e52287. doi: 10.2196/52287 (PMC11502985; doi:10.2196/52287)
Supplement: Multimedia Appendix 2 [file ijmr_v13i1e52287_app2.docx]

| **Search Step** | **Description** |
| --- | --- |
| Query Terms | Quotations were used when searching for terms with more than one word  “Health Locus of Control”; “Behavior Change”; “Behavioral Change”; “Behavioral Change Interventions”; “Medication Adherence”; “HLOC”; “Patients”; “Healthcare; “Culture”; “Empirical”; and “Locus of Control”  British spelling of the world Behaviour was also used as a search term for the following: “Behaviour Change”; “Behavioural Change”: “Behavioural Change Interventions” |
| Filters and Limits | Date: January 1960 – December 2022  Outlets: Web of Science, IEEE Xplore, ACM Digital Library, AIS e-Library, PubMed  Languages: English  Publication Type: Peer-reviewed Journals |
| Search Strings | The term Health Locus of Control was always included in search strings. Quotations were used when searching for terms containing more than one word. Search strings contained the phrase “Health Locus of Control” and one of the terms below. For example:  “Health Locus of Control” AND “Healthcare”  “Health Locus of Control” OR “Medication Adherence”  In searches where the term “Behavior” was included, the spellings of “Behavior” and “Behaviour” were used with the keyword OR. For example:  “Health Locus of Control” AND (“Behavior Change” OR “Behavioural Change”)  20 search strings in total were used. The list of terms used are as follows:  “Health Locus of Control” AND:  “Behavior Change”; “Behavioral Change”; “Behavioral Change Interventions”; “Medication Adherence”; “HLOC”; “Patients”; “Healthcare; “Culture”; “Empirical”; and “Locus of Control”.  “Health Locus of Control” OR:  “Behavior Change”; “Behavioral Change”; “Behavioral Change Interventions”; “Medication Adherence”; “HLOC”; “Patients”; “Healthcare; “Culture”; “Empirical”; and “Locus of Control”.  This search strategy was used for all five outlets searched. |
