# Supplementary material for: Health Locus of Control and Medical Behavioral Interventions: Systematic Review and Recommendations
Source: Interact J Med Res. 2024 Oct 10;13:e52287. doi: 10.2196/52287 (PMC11502985; doi:10.2196/52287)
Supplement: Multimedia Appendix 3 [file ijmr_v13i1e52287_app3.docx]

| **#** | **Citation** | **Study Type** | **Sample Size** | **Interaction** |
| --- | --- | --- | --- | --- |
|  | (Ahadzadeh et al., 2021) | Survey | 374 | HLOC Constructs mediate demographic characteristics and intention to adopt mHealth Interventions |
|  | (Bennett et al., 2017) | Survey | 276 | Higher Internal LOC leads to more willingness to use health applications and online trackers.  Higher Power Others LOC leads to more willingness to use mHealth |
|  | (Bianchi et al., 2022) | Survey | 188 | Higher chance LOC leads to less preventative health behaviors (“it doesn’t matter what I do…”)  Higher powerful others HLOC leads to more preventative behaviors (Physicians, officials, authority) |
|  | (Ganjoo et al., 2021) | Survey | 250 | Higher Internal LOC leads to lower perceived stress.  Higher Chance and Powerful others HLOC leads to higher perceived stress |
|  | (Lee et al., 2021) | Survey | 844 | Sports participation leads to higher internal LOC among adolescents |
|  | (Mercer et al., 2018) | Survey | 599 | Higher I LOC leads to more leisure time.  Lower I LOC leads to less alcohol consumption.  Chance HLOC leads to higher likelihood of smoking.  PO LOC leads to lower likelihood of smoking |
|  | (Kesavayuth et al., 2020) | Archival Data Analysis | 16,284 | Higher Internal LOC leads to better physical and mental health.  Higher internal LOC leads to more social behaviors, which then leads to positive health influence (Mediation between social behaviors, HLOC, and health behavior constructs) |
|  | (Musich et al., 2020) | Survey | 3934 | Higher Internal LOC leads to better healthcare outcomes across income levels.  Social contact leads to better healthcare behaviors across income levels  Health information-seeking behavior is linked to higher Internal LOC |
|  | (Shin & Lee, 2021) | Survey | 600 | Higher Internal LOC leads to lower propensity of mental problems.  Mental health issues mediate the effect  Additional factors such as age, marriage status, income, and religion can play a factor in mental-well being |
|  | (Imeri et al., 2022) | Survey | 300 | Patients with chronic conditions tend to have higher Chance LOC  High Powerful others LOC and activation is negative.  Internal HLOC and activation is negative relationship |
|  | (Duplaga & Grysztar, 2021) | Survey | 2223 | Higher health locus leads to less consumption of unhealthy foods, more consumption of healthy foods.  Higher Powerful Others LOC led to more beneficial dietary patterns (parents as likely powerful others)  Higher Chance LOC leads to less beneficial dietary patterns |
|  | (Wang et al., 2022) | Survey | 254 | Chance LOC has a moderating effect on patient Self-efficacy and self-management behavior.  High self-efficacy leads to more self-management |
|  | (Mehta & Narayanan, 2021) | Survey | 170 | Positive emotional reactivity is predictive of higher internal LOC  Negative emotional reactivity is predictive of higher Chance and Power Others LOC |
|  | (Marton et al., 2021) | Survey | 153 | Doctors are a power other when operationalizing Powerful Others LOC |
|  | (Schreitmüller & Loerbroks, 2020) | Survey | 205 | Higher Self-efficacy and Internal LOC is associated with better asthma control.  Higher External LOC beliefs were associated with poorer asthma control |
|  | (Boyd & Wilcox, 2020) | Survey | 838 | Exploration of belief in God as a LOC  Positive relationship between GLOC, Chance LOC, and PO LOC |
|  | (Aviad-Wilchek, 2021) | Survey | 124 | Higher Internal LOC > Less suicidal tendencies  Higher Internal LOC > Higher meaning of life  Mental illness > more external LOC, lower meaning of life, higher suicidal tendency |
|  | (Wrightson & Wardle, 1997) | Survey | 128 | Exploration of cultural differences in HLOC constructs.  South Asian females scored higher on Chance LOC and PO LOC |
|  | (Reknes et al., 2019) | Archival Data Analysis | 1474 | Higher external LOC moderates bullying behaviors and mental strain, higher external LOC decreases mental strain from bullying |
|  | (Olagoke et al., 2021) | Survey | 501 | Religion and faith is positively correlated with trust in informal sources of information  Higher religiosity has a negative association with intention to vaccinate |
|  | (Mahmoud et al., 2022) | Survey | 847 | HLOC moderates between anxiety and alienation in job-related stresses.  Not directly related to health behaviors, more on psychology and stress |
|  | (Helmer et al., 2012) | Survey | 3,306 | Direct relationships between higher I-HLOC and preventive health behaviors such as exercise and dieting. Higher PO HLOC directed to physicians increased doctor-prescribed behaviors |
|  | (Amit Aharon et al., 2018) | Survey | 731 | High PO-HLOC > vaccination compliance  High I-HLOC and C-HLOC > indirect associations regarding vaccines |
|  | (Morishita et al., 2017) | Survey | 173 | External HLOC constructs accounted for higher rates of self-care amongst elderly patients. External HLOC sources included family members and spirituality. |
|  | (Chandra & Yagnik, 2022) | Experiment |  | HLOC <> Stress |
|  | (Hillen et al., 2014) | Survey | 345 | Higher E-HLOC > more trust |
|  | (Akanni & Koleoso, 2022) | Survey | 317 | Students, mean age 23.8.  External LOC affects death-related anxiety.  Death is observed as uncontrollable, higher external LOC leads to less stress regarding the uncontrollable nature of death |
|  | (Brown et al., 2017) | Survey | 100 | Multi-metric study on terminally ill cancer patients  Higher internal LOC can lead to more adverse health outcomes (physical and mental) at end of life.  Observation: higher internal LOC is challenging in a terminal state, less control over life and inevitability of death |
|  | (Halse et al., 2021) | Survey and Experiment | 52 | Dementia patients, 12-month study  All patients progressed in dementia during duration of study.  LOC became more external as symptoms progressed.  However, higher external LOC was observed with lower patient depression.  External LOC could be linked to acceptance of a chronic condition. |
|  | (Kaynak et al., 2022) | Survey | 422 | Study on trait anxiety, behavioral inhibitions, and LOC  LOC predicts trait anxiety and behavioral inhibitions.  Lower behavioral inhibitions is linked to external LOC  External LOC is linked to higher anxiety levels (lack of personal control) |
|  | (Krampe et al., 2021) | Multiple Survey | 2752 | Norwegian and German sampling  LOC plus COVID-19 related stress and general mental stress  Internal LOC decreased relationship between COVID-19 Stress and general stress (Moderating effect)  External LOC increase relationship, also as moderator |
|  | (Lima et al., 2022) | Survey | 220 | Cancer outpatients  PO LOC mediates between big-5 personality traits and depression symptoms.  Extroversion leads to external support and Conscientiousness leads to more reliance on physician recommendations.  Both linked to PO LOC, both have positive effects on patient well-being |
|  | (Micheletto et al., 2022) | Survey | 666 | Cross-sectional study over 2 years before and during covid pandemic  Study on personal growth, I LOC, and optimism  Internal LOC has direct and positive association with personal growth.  Internal LOC has an indirect effect (mediated by personal growth) with happiness |
|  | (Mori et al., 2022) | Survey | 1430 | Japanese residents, stress during COVID-19 and LOC  Higher Internal LOC led to decreased stress.  External LOC led to increased stress after lifestyle changes from the pandemic.  Increasing Internal LOC and decreasing External LOC can lead to less stress in COVID context |
|  | (Sharif, 2017) | Survey | 118 | Breast cancer patients  Locus of control, anxiety, uncertainty, depression  Higher internal LOC and lower external LOC leads to higher quality of life, lower anxiety, and lower depression.  Uncertainty mediates between LOC and quality of life (weak relationship)  Note: Findings are reversed for terminally ill patients, may indicate that while there is hope of recovery higher internal LOC leads to better mental state |
|  | (Tsiouli et al., 2014) | Randomized Control Trial | 44 | Parenting stress, perceived stress, relaxation techniques  8-week trial 19 intervention group (relaxation exercises) 25 control group  Control group increased Chance HLOC, which led to more anxiety and stress.  Intervention group had improved metrics and decreased stress |
|  | (Weinhardt & Ruckert, 2022) | Survey | 422 | Personal control, pro-environmental behavior in COVID-19 context  Internal LOC led to higher protective health-related behaviors and more subject proactivity |
|  | (Würtzen et al., 2022) | Survey | 5560 | Mental well-being, general health, locus of control  COVID-19 led to decreased internal locus of control and decreased mental well-being.  Effect is pronounced for individuals under 30. |
|  | (Aviad & Cohen-Louck, 2021) | Questionnaires | 195 | LOC in older adults, Risk for suicide in older adults  Negative correlation between purpose in life and risk for suicide  Negative correlation between internal locus of control and the risk for suicide  Positive correlation between internal locus of control and the level of purpose in life |
|  | (Gore et al., 2016) | Surveys | 2029 | Perceived constraints related to External LOC were a consistent predictor of physical and mental well-being among undergraduate college students.  Internal LOC and External LOC suggested to be separated when measuring well-being associated with internal beliefs. |
|  | (Kuwahara et al., 2004) | Questionnaires | 4842 | MHLC scores were correlated with health-related behavior.  The use of the MHLC scale   - Should help to provide a better understanding of Japanese health belief, - Could be beneficial for developing effective health education programs |
|  | (Grotz et al., 2011) | Survey | 5542 | Sociodemographic correlates with HLC and the association between HLC and health behavior.  Higher values on the internal and powerful others dimension 🡪 Men  Higher powerful others and chance HLC 🡪 Higher Age  The risk of unhealthy behavior 🡪 similar 🡪 persons with higher HLC (Does not apply for chance HLOC) |
|  | (Moshki et al., 2012) | Experiment | 144 | Evaluation of LOC and Self-Esteem to determine responses to problems related to mental health and potential responses to LOC and Self-esteem constructs.  Promotion of mental health (intervention) increased I LOC and self-esteem. Student mental health also increased. E LOC decreased after intervention. |
|  | (Holroyd et al., 2001) | Survey | 290 | mental health symptoms, health-related behaviors, health locus of control and social support  Internal locus of control 🡪 be well informed about what is happening to them and more likely to take action to prevent the onset of illness  low internal locus of control contributes to female immigrant domestic helpers being distinguished by characteristics of powerlessness such as withdrawal and passivity  Effects of level of LOC is examined in the survey. |
|  | (Cohen & Azaiza, 2007) | Survey | 358 & 162 | external HLC, balanced nutrition, and attending regular checkups varied by ethnicity.  Lower internal HLC and lower engagement in physical activity >> Arab women  Jewish ethnicity and male sex >> sig with Internal HLOC  Arab ethnicity, older age and lower education >> sig external HLOC  higher internal HLC explained the variance of balanced nutrition |
|  | (Pieterse & Carter, 2010) | Survey | 90 | Experiences of racism were associated with perceptions of health status and health locus of control.  Racial identity attitudes were associated with health locus of control.  HLOC and health behavior must be studied |
|  | (Egan et al., 2009) | Interview | 3665 | American Indians  Higher chance HLOC in women  Similar MHLOC in both men and women  Lower Internal HLOC >> Higher age  Lower PO HLOC >> Higher Age  Higher education >> Lower chance HLOC |
|  | (van Dijk et al., 2013) | Experiment | 10302 | HLC contributes to ethnic differences in the prevalence of depressive symptoms.  Higher external LOC 🡪 more likely to have depressive symptoms.  Higher internal LOC 🡪 Less likely to have depressive symptoms. |
|  | (Iskandarsyah et al., 2014) | Survey | 120 | HLC among women with breast cancer and healthy women in the Indonesian population  Women with breast cancer:   - Higher external HLC - Lower internal HLC - God LHC explained a significant amount of the variance of anxiety, - None of HLC orientations was related to depressive symptoms.   Women with breast cancer tend to attribute their illness to external sources of control.  The perceived uncontrollable nature of cancer and the uncertainty of treatment outcomes might reduce patients’ beliefs in personal control over their illness. |
|  | (Poortinga et al., 2008) | Survey | 10,892 | Examination through multilevel modeling to identify HLC beliefs and whether a mediating effect exists between regional socio-economic status and health effects  HLC beliefs mediated partially between socio-economic status and individual health and well-being |

Reference List

Ahadzadeh, A. S., Wu, S. L., Ong, F. S., & Deng, R. (2021). The Mediating Influence of the Unified Theory of Acceptance and Use of Technology on the Relationship Between Internal Health Locus of Control and Mobile Health Adoption: Cross-sectional Study. *Journal of Medical Internet Research*, *23*(12), e28086.

Akanni, O., & Koleoso, O. (2022). Self-esteem, locus of control and religiosity in predicting death anxiety among students in a Nigerian tertiary institution. *Journal of Clinical and Scientific Research*, *11*(1), 28–28.

Amit Aharon, A., Nehama, H., Rishpon, S., & Baron-Epel, O. (2018). A path analysis model suggesting the association between health locus of control and compliance with childhood vaccinations. *Human Vaccines & Immunotherapeutics*, *14*(7), 1618–1625.

Aviad, Y., & Cohen-Louck, K. (2021). Locus of Control and Purpose in Life as Protective Factors against the Risk for Suicide in Older Adults. *Smith College Studies in Social Work*, *91*(4), 295–308. https://doi.org/10.1080/00377317.2021.1968323

Aviad-Wilchek, Y. (2021). Locus of control and the meaning of life as a salutogenic model that reduces suicidal tendencies in patients with mental illness. *Current Psychology*, *40*(2), 465–474. https://doi.org/10.1007/s12144-018-0122-2

Bennett, B. L., Goldstein, C. M., Gathright, E. C., Hughes, J. W., & Latner, J. D. (2017). Internal health locus of control predicts willingness to track health behaviors online and with smartphone applications. *Psychology, Health & Medicine*, *22*(10), 1224–1229.

Bianchi, D., Lonigro, A., Norcia, A. D., Tata, D. D., Pompili, S., Zammuto, M., Cannoni, E., Longobardi, E., & Laghi, F. (2022). A model to understand COVID-19 preventive behaviors in young adults: Health locus of control and pandemic-related fear. *Journal of Health Psychology*, *27*(14), 3148–3163. https://doi.org/10.1177/13591053221089722

Boyd, J. M., & Wilcox, S. (2020). Examining the relationship between health locus of control and God Locus of Health Control: Is God an internal or external source? *Journal of Health Psychology*, *25*(7), 931–940. https://doi.org/10.1177/1359105317739099

Brown, A. J., Thaker, P. H., Sun, C. C., Urbauer, D. L., Bruera, E., Bodurka, D. C., & Ramondetta, L. M. (2017). Nothing left to chance? The impact of locus of control on physical and mental quality of life in terminal cancer patients. *Supportive Care in Cancer*, *25*(6), 1985–1991. https://doi.org/10.1007/s00520-017-3605-z

Chandra, Y., & Yagnik, J. (2022). Experience of Perceived Stress and Impact of Health Locus of Control During COVID-19 Pandemic: Investigating Entrepreneurs and Corporate Employees. *South Asian Journal of Human Resources Management*, 23220937221076245.

Cohen, M., & Azaiza, F. (2007). Health-Promoting Behaviors and Health Locus of Control from a Multicultural Perspective. *Ethnicity & Disease*, *17*(4), 636–642.

Duplaga, M., & Grysztar, M. (2021). Nutritional Behaviors, Health Literacy, and Health Locus of Control of Secondary Schoolers in Southern Poland: A Cross-Sectional Study. *Nutrients*, *13*(12), Article 12. https://doi.org/10.3390/nu13124323

Egan, J. T., Leonardson, G., Best, L. G., Welty, T., Calhoun, D., & Beals, J. (2009). Multidimensional Health Locus of Control in American Indians: The Strong Heart Study. *Ethnicity & Disease*, *19*(3), 338–344.

Ganjoo, M., Farhadi, A., Baghbani, R., Daneshi, S., & Nemati, R. (2021). Association between health locus of control and perceived stress in college student during the COVID-19 outbreak: A cross-sectional study in Iran. *BMC Psychiatry*, *21*(1), 529. https://doi.org/10.1186/s12888-021-03543-1

Gore, J. S., Griffin, D. P., & McNierney, D. (2016). Does Internal or External Locus of Control Have a Stronger Link to Mental and Physical Health? *Psychological Studies*, *61*(3), 181–196. https://doi.org/10.1007/s12646-016-0361-y

Grotz, M., Hapke, U., Lampert, T., & Baumeister, H. (2011). Health locus of control and health behaviour: Results from a nationally representative survey. *Psychology, Health & Medicine*, *16*(2), 129–140. https://doi.org/10.1080/13548506.2010.521570

Halse, I., Bjørkløf, G. H., Engedal, K., Selbæk, G., & Barca, M. L. (2021). One-Year Change in Locus of Control among People with Dementia. *Dementia and Geriatric Cognitive Disorders Extra*, *11*(3), 298–305. https://doi.org/10.1159/000520248

Helmer, S. M., Krämer, A., & Mikolajczyk, R. T. (2012). Health-related locus of control and health behaviour among university students in North Rhine Westphalia, Germany. *BMC Research Notes*, *5*(1), 703. https://doi.org/10.1186/1756-0500-5-703

Hillen, M. A., de Haes, H. C., Stalpers, L. J., Klinkenbijl, J. H., Eddes, E.-H., Verdam, M. G., & Smets, E. M. (2014). How attachment style and locus of control influence patients’ trust in their oncologist. *Journal of Psychosomatic Research*, *76*(3), 221–226.

Holroyd, E. A., Molassiotis, A., & Taylor-Pilliae, R. E. (2001). Filipino Domestic Workers in Hong Kong: Health Related Behaviors, Health Locus of Control and Social Support. *Women & Health*, *33*(1–2), 181–205. https://doi.org/10.1300/J013v33n01_11

Imeri, H., Holmes, E., Desselle, S., Rosenthal, M., & Barnard, M. (2022). A survey study of adults with chronic conditions: Examining the correlation between patient activation and health locus of control. *Chronic Illness*, 17423953211067431. https://doi.org/10.1177/17423953211067431

Iskandarsyah, A., de Klerk, C., Suardi, D. R., Sadarjoen, S. S., & Passchier, J. (2014). Health Locus of Control in Indonesian Women with Breast Cancer: A Comparison with Healthy Women. *Asian Pacific Journal of Cancer Prevention*, *15*(21), 9191–9197. https://doi.org/10.7314/APJCP.2014.15.21.9191

Kaynak, H., Turan, A., & Demir, Y. (2022). Locus of Control as a Mediator of the Relationships Between Motivational Systems and Trait Anxiety. *Psychological Reports*, 00332941221139707. https://doi.org/10.1177/00332941221139707

Kesavayuth, D., Poyago-Theotoky, J., Tran, D. B., & Zikos, V. (2020). Locus of control, health and healthcare utilization. *Economic Modelling*, *86*(C), 227–238.

Krampe, H., Danbolt, L. J., Haver, A., Stålsett, G., & Schnell, T. (2021). Locus of control moderates the association of COVID-19 stress and general mental distress: Results of a Norwegian and a German-speaking cross-sectional survey. *BMC Psychiatry*, *21*(1), 437. https://doi.org/10.1186/s12888-021-03418-5

Kuwahara, A., Nishino, Y., Ohkubo, T., Tsuji, I., Hisamichi, S., & Hosokawa, T. (2004). Reliability and Validity of the Multidimensional Health Locus of Control Scale in Japan: Relationship with Demographic Factors and Health-Related Behavior. *The Tohoku Journal of Experimental Medicine*, *203*(1), 37–45. https://doi.org/10.1620/tjem.203.37

Lee, D.-J., So, W.-Y., & Lee, S.-M. (2021). The Relationship between Korean Adolescents’ Sports Participation, Internal Health Locus of Control, and Wellness during COVID-19. *International Journal of Environmental Research and Public Health*, *18*(6), Article 6. https://doi.org/10.3390/ijerph18062950

Lima, M. P., Moret-Tatay, C., & Irigaray, T. Q. (2022). Locus of control, personality and depression symptoms in cancer: Testing a moderated mediation model. *Clinical Psychology & Psychotherapy*, *29*(2), 489–500. https://doi.org/10.1002/cpp.2604

Mahmoud, A. B., Reisel, W. D., Fuxman, L., & Hack-Polay, D. (2022). Locus of control as a moderator of the effects of COVID-19 perceptions on job insecurity, psychosocial, organisational, and job outcomes for MENA region hospitality employees. *European Management Review*, *19*(2), 313–332. https://doi.org/10.1111/emre.12494

Marton, G., Pizzoli, S. F. M., Vergani, L., Mazzocco, K., Monzani, D., Bailo, L., Pancani, L., & Pravettoni, G. (2021). Patients’ health locus of control and preferences about the role that they want to play in the medical decision-making process. *Psychology, Health & Medicine*, *26*(2), 260–266. https://doi.org/10.1080/13548506.2020.1748211

Mehta, R., & Narayanan, M. (2021). *The Relationship of Emotional Reactivity with Health Locus of Control*. *16*, 47–56.

Mercer, D. A., Ditto, B., Lavoie, K. L., Campbell, T., Arsenault, A., & Bacon, S. L. (2018). Health Locus of Control Is Associated With Physical Activity and Other Health Behaviors in Cardiac Patients. *Journal of Cardiopulmonary Rehabilitation and Prevention*, *38*(6), 394. https://doi.org/10.1097/HCR.0000000000000350

Micheletto, V., Zito, M., Bustreo, M., Gabrielli, G., Circi, R., & Russo, V. (2022). The Impact of Optimism and Internal Locus of Control on Workers’ Well-Being, A Multi-Group Model Analysis before and during the COVID-19 Pandemic. *Social Sciences*, *11*(12), Article 12. https://doi.org/10.3390/socsci11120559

Mori, M., Seko, T., & Ogawa, S. (2022). Association of Social Capital and Locus of Control with Perceived Health during the COVID-19 Pandemic in Japan. *International Journal of Environmental Research and Public Health*, *19*(15), Article 15. https://doi.org/10.3390/ijerph19159415

Morishita, M., Hattori, S., & Miyai, N. (2017). Ability for Self-Care among Elderly Patients with Diabetes Mellitus and Its Association with Health Locus of Control and Social Support. *Nihon eiseigaku zasshi Japanese journal of hygiene*, *72*(1), 77–86. https://doi.org/10.1265/jjh.72.77

Moshki, M., Amiri, M., & Khosravan, S. (2012). Mental health promotion of Iranian university students: The effect of self-esteem and health locus of control. *Journal of Psychiatric and Mental Health Nursing*, *19*(8), 715–721. https://doi.org/10.1111/j.1365-2850.2011.01806.x

Musich, S., Wang, S. S., Slindee, L., Kraemer, S., & Yeh, C. S. (2020). The impact of internal locus of control on healthcare utilization, expenditures, and health status across older adult income levels. *Geriatric Nursing*, *41*(3), 274–281. https://doi.org/10.1016/j.gerinurse.2019.10.008

Olagoke, A. A., Olagoke, O. O., & Hughes, A. M. (2021). Intention to Vaccinate Against the Novel 2019 Coronavirus Disease: The Role of Health Locus of Control and Religiosity. *Journal of Religion and Health*, *60*(1), 65–80. https://doi.org/10.1007/s10943-020-01090-9

Pieterse, A. L., & Carter, R. T. (2010). An Exploratory Investigation of the Relationship between Racism, Racial Identity, Perceptions of Health, and Health Locus of Control among Black American Women. *Journal of Health Care for the Poor and Underserved*, *21*(1), 334–348. https://doi.org/10.1353/hpu.0.0244

Poortinga, W., Dunstan, F. D., & Fone, D. L. (2008). Health locus of control beliefs and socio-economic differences in self-rated health. *Preventive Medicine*, *46*(4), 374–380.

Reknes, I., Visockaite, G., Liefooghe, A., Lovakov, A., & Einarsen, S. V. (2019). Locus of Control Moderates the Relationship Between Exposure to Bullying Behaviors and Psychological Strain. *Frontiers in Psychology*, *10*. https://www.frontiersin.org/articles/10.3389/fpsyg.2019.01323

Schreitmüller, J., & Loerbroks, A. (2020). The role of self-efficacy and locus of control in asthma-related needs and outcomes: A cross-sectional study. *Journal of Asthma*, *57*(2), 196–204. https://doi.org/10.1080/02770903.2018.1556687

Sharif, S. P. (2017). Locus of control, quality of life, anxiety, and depression among Malaysian breast cancer patients: The mediating role of uncertainty. *European Journal of Oncology Nursing*, *27*, 28–35.

Shin, S., & Lee, E. (2021). Relationships among the Internal Health Locus of Control, Mental Health Problems, and Subjective Well-Being of Adults in South Korea. *Healthcare*, *9*(11), Article 11. https://doi.org/10.3390/healthcare9111588

Tsiouli, E., Pavlopoulos, V., Alexopoulos, E. C., Chrousos, G., & Darviri, C. (2014). Short-Term Impact of a Stress Management and Health Promotion Program on Perceived Stress, Parental Stress, Health Locus of Control, and Cortisol Levels in Parents of Children and Adolescents With Diabetes Type 1: A Pilot Randomized Controlled Trial. *EXPLORE*, *10*(2), 88–98. https://doi.org/10.1016/j.explore.2013.12.004

van Dijk, T. K., Dijkshoorn, H., van Dijk, A., Cremer, S., & Agyemang, C. (2013). Multidimensional health locus of control and depressive symptoms in the multi-ethnic population of the Netherlands. *Social Psychiatry and Psychiatric Epidemiology*, *48*(12), 1931–1939. https://doi.org/10.1007/s00127-013-0678-y

Wang, R., Zhou, C., Wu, Y., Sun, M., Yang, L., Ye, X., & Zhang, M. (2022). Patient empowerment and self-management behaviour of chronic disease patients: A moderated mediation model of self-efficacy and health locus of control. *Journal of Advanced Nursing*, *78*(4), 1055–1065. https://doi.org/10.1111/jan.15077

Weinhardt, C. B., & Ruckert, J. H. (2022). Internal Locus of Control Predicts Proenvironmental and COVID-19 Health-Related Behaviors: A Pilot Study. *Ecopsychology*. https://doi.org/10.1089/eco.2022.0016

Wrightson, K. J., & Wardle, J. (1997). Cultural variation in health locus of control. *Ethnicity & Health*, *2*(1–2), 13–20. https://doi.org/10.1080/13557858.1997.9961811

Würtzen, H., Clausen, L. H., Andersen, P. B., Santini, Z. I., Erkmen, J., & Pedersen, H. F. (2022). Mental well-being, health, and locus of control in Danish adults before and during COVID-19. *Acta Neuropsychiatrica*, *34*(2), 93–98. https://doi.org/10.1017/neu.2021.37
